# Effect of the CP@clinic program (Community Paramedicine at Clinic program) on health care utilization from administrative data during and after the intervention: a cluster-randomized controlled trial

**DOI:** 10.3389/frhs.2026.1939050

**Published:** 2026-10-15

**Authors:** Gina Agarwal, Melissa Pirrie, Manasvi Sai Vanama, Zheng Jing Hu, J. Michael Paterson, Lehana Thabane, Ricardo Angeles, Francine Marzanek

**Affiliations:** 1 Department of Family Medicine, McMaster University, Hamilton, ON, Canada; 2 Department of Health Research Methods, Evidence, and Impact, McMaster University, Hamilton, ON, Canada; 3 ICES, Toronto, ON, Canada; 4 St Joseph’s Health Care Hamilton, Hamilton, ON, Canada; 5 Institute of Health Policy, Management and Evaluation, University of Toronto, Toronto, ON, Canada

**Keywords:** community paramedicine, health service utilization, hospital admissions, primary care, social housing

## Abstract

**Introduction:**

The study objective was to compare the effects of the Community Paramedicine at Clinic (CP@clinic) program versus usual care on building-level health service utilization during a 12-month trial period and 12 months post-trial.

**Methods:**

This study employed a longitudinal, repeated cross-sectional design within a cluster-randomized controlled trial (RCT). It involved 30 apartment buildings across five Ontario communities, housing low-income older adults. Buildings were paired and randomized to receive either the CP@clinic intervention or continuing with usual care. Data was derived from ICES-maintained administrative health databases, linked using unique identifiers. The sample included all residents aged 55 + in the study postal codes from the Ontario Health Insurance Plan (OHIP) Registered Persons Database (RPDB), excluding those aged 120+, ineligible for OHIP, and those with no health care encounters within 7 years. The primary health care utilization outcome was emergency department visits by ambulance and other outcomes included hospital admissions and primary care visits.

**Results:**

CP@clinic buildings showed significantly lower hospital admission rates during both the 12-month RCT period (adjusted incidence rate ratio [aIRR]: 0.75 [0.61,0.91]) and the 12-month post-RCT period [aIRR: 0.82 (0.70,0.96)] compared to usual care buildings. Primary care visits were lower in CP@clinic buildings during the RCT (aIRR: 0.93 [0.88,0.99]) but higher in the post-RCT period (aIRR: 1.05 [1.00,1.10]). No significant differences were found in emergency department visits or the number of days admitted between groups in either period.

**Discussion:**

CP@clinic was linked to a sustained reduction in hospital admissions and an increase in primary care visits beyond the RCT time period, reflecting a shift from reactive to preventive care among older adults residing in social housing. The lasting impact beyond the RCT period suggests that the CP@clinic Program can improve health system efficiency, primary care access, and reduce health inequities in vulnerable populations.

**Trial registration:**

ClinicalTrials.gov, Identifier: NCT02152891

## 1 Introduction

Older adults in Canada represent a growing proportion of the population, with those in lower-income brackets experiencing greater health disparities, including higher rates of chronic diseases, multimorbidity, and increased mortality (1). These individuals often encounter barriers to accessing primary care, leading to a greater reliance on emergency medical services (EMS) for conditions that could otherwise be managed through preventive health interventions (2). Addressing these challenges requires innovative, community-based programs that promote equitable health access and reduce the strain on emergency care systems. Community paramedicine is one such adaptation whereby paramedics embedded in the community cater towards non-acute health-related problems (3).

The Community Paramedicine at Clinic (CP@clinic) Program is an evidence-based community health initiative designed to improve health outcomes in social housing settings (4). Evaluated through a randomized controlled trial (RCT), CP@clinic was associated with a reduction of 0.90 EMS calls per month per 100 apartment units across five communities in Ontario, Canada (5, 6). Additionally, the program demonstrated improvements in health-related quality of life and reductions in chronic disease risk factors among residents (5, 6). The RCT also showed that CP@clinic increased connections to home care services, increased primary care visits, and reduced transfers to long-term care from social housing (7). Although CP@clinic was shown to have an impact on health care utilization during the trial, its effects on health care utilization following the trial, when paramedic services were not following the RCT protocol, and pragmatically continued a “usual community wellness clinic care model”, are not known. This study examines whether the reduction in EMS calls and improvements in health outcomes observed during the RCT were sustained in the year following program cessation.

The objective of this study was to compare the effects of CP@clinic versus usual care on health service utilization during the 12-month RCT period and 12 months post-RCT. We hypothesized that the program's impact on EMS and broader health service utilization would be sustained beyond the 12-month RCT period. Findings from this study can contribute to evidence on the impacts of paramedicine-led health initiatives and inform future policy on the scalability and duration of such programs.

## 2 Materials & methods

### 2.1 Study design

This is a longitudinal study of an open-label, cluster-randomized controlled trial with paired randomization at the social housing building level. It includes 30 apartment buildings across five Ontario communities (Figure 1). The buildings were home to low-income seniors, with rent capped at 30% of household income and the remainder subsidized by the government. Detailed methods and the RCT have been published elsewhere (7, 8).

**Figure 1:**
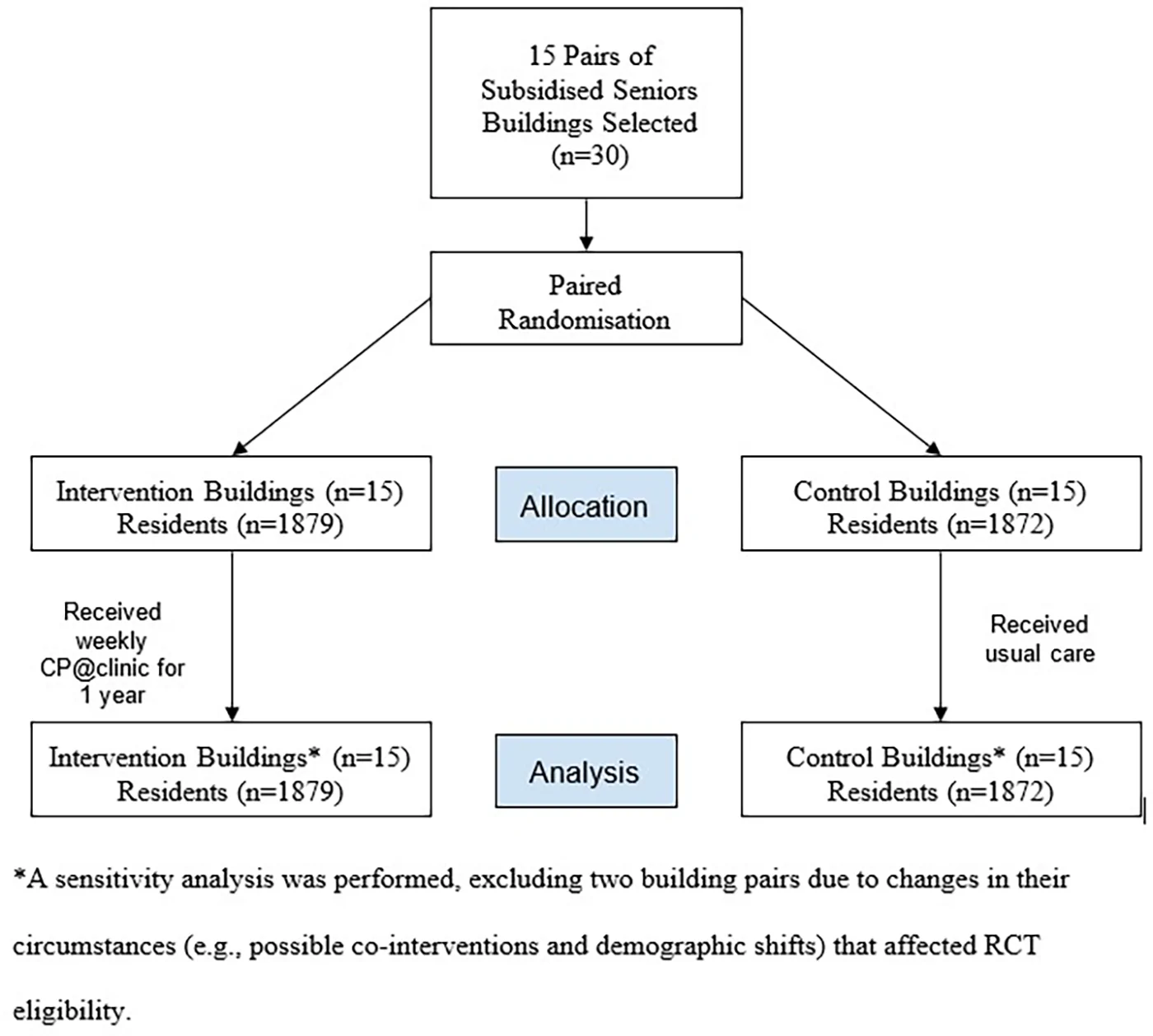
CONSORT flow diagram of the CP@clinic RCT.

Approval was granted by the Hamilton Integrated Research Ethics Board, which waived the requirement for individual resident consent for the creation of building-level resident cohorts using administrative health care data at ICES (formerly known as the Institute for Clinical Evaluative Sciences) (9). Residents in the CP@clinic program provided written informed consent for linking the CP@clinic assessment data they provided with the administrative data held at ICES. ICES, an independent nonprofit research institute funded by the Ontario Ministry of Health which is authorized under Ontario's health privacy law to collect and analyze health data for research purposes under a Research Ethics Board-approved research protocol. This report follows the CONSORT (10) guidelines and has been registered on clinicaltrials.gov.

### 2.2 Intervention

The CP@clinic intervention was a community paramedicine program focused on health promotion and disease prevention. Each week, two specially trained paramedics offered a voluntary, half-day drop-in session for residents in a common area of the social housing building. During these sessions, paramedics conducted health risk assessments using validated tools including cardiometabolic risk screening and social determinants of health questionnaires. Based on the assessment, paramedics worked with residents to address key risk factors, providing tailored education, goal setting, and referrals to community resources such as homecare services. Residents were also encouraged to revisit CP@clinic sessions to monitor progress, and address challenges and additional risk factors. For those with a family physician, assessment results were shared with their physician after obtaining the resident's consent.

The intervention was implemented across multiple sites, each for a 12-month period. Sites had staggered start dates, with all interventions taking place between July 1, 2015, and November 30, 2016. For the 30 sites: eight sites ran from January 1 to December 31, 2015; 14 sites from February 1, 2015, to January 31, 2016; two sites from July 1, 2015, to June 30, 2016; two sites from August 1, 2015, to July 31, 2016; and four sites from December 1, 2015, to November 30, 2016. The period of follow-up for this study was the 12 months immediately following cessation of the RCT period for each site (also staggered).

While intervention delivery was once a week across all sites for the RCT period, during the post-RCT period, the intervention was delivered once a week by 3 services, and dropped to once a month and once every 6 weeks for the other two services. However, participation rates were stable between the intervention and post-intervention periods.

### 2.3 Study participants

The intervention was available to all building residents, randomly selected to receive the CP@clinic intervention, where the buildings served as the unit of randomization. The building-level inclusion criteria were: (1) a unique postal code not shared with neighbouring residences; (2) at least 50 apartment units; (3) at least 60% of residents aged 55 years or older; and (4) presence of another building of similar size, demographic characteristics, and geographic location to permit paired randomization. Using an intention-to-treat approach, all residents aged 55 + years in the study postal codes each month, as per the Ontario Health Insurance Program (OHIP) Registered Persons Database (RPDB), were included in the monthly building-level data. Exclusions were made for individuals aged 120 + years according to the RPDB, those ineligible for OHIP at the time of the trial, and those with no health care encounters in the preceding seven years (as a proxy for unrecorded emigration).

A *post-hoc* sensitivity analysis was conducted excluding two intervention buildings and their matched comparators because the intervention buildings were deemed to be ineligible partway through the intervention. The first excluded building was originally believed to have a distinct postal code but was later found to be part of a larger complex that included assisted-living units sharing the same postal code. The second building was excluded as it experienced a surge in neighborhood crime and EMS calls following the closure of a nearby police station, making it no longer comparable to its matched usual care site, as many older residents became hesitant to leave their homes.

### 2.4 Randomization and masking

Intervention and control buildings were paired based on similar characteristics in collaboration with municipal housing providers (see prior publication) (7). Using a computer-generated randomization tool, one building from each pair was randomly assigned to receive the CP@clinic intervention and the other to usual care. Due to the nature of the CP@clinic drop-in program, the masking of residents was not feasible. However, residents in the CP@clinic buildings were not informed of the details of the trial outcome measures, and usual care building residents were not explicitly informed they were in the control group.

### 2.5 Procedures

To establish the building-level cohort for the intention-to-treat analysis, postal codes of participating social housing buildings were shared with ICES, and the corresponding demographic and health care records of residents were retrieved and securely analyzed at ICES using records linked at the person level using unique, encoded identifiers.

#### 2.5.1 Demographic characteristics and health status

Resident age and sex at the RCT start date were obtained from the OHIP RPDB (11); comprehensive data on race or ethnicity were not available. Frailty was assessed using the Johns Hopkins Adjusted Clinical Groups (ACG) Frailty Indicator (from the Johns Hopkins ACG® System, Version 10) (12).

#### 2.5.2 Health service utilization outcomes

Health service utilization was assessed over three periods: 1 month prior to the RCT, the 12-month RCT period, and the 12 months following the RCT. Data on the number of unplanned emergency department (ED) visits, both general and those involving ambulance transport, were sourced from the Canadian Institute for Health Information (CIHI) National Ambulatory Care Reporting System (13). Non-elective hospital admissions (urgent or emergent) were identified in the CIHI Discharge Abstract Database (14) using the hospital admission type variable applying an episode-of-care method that grouped inter-hospital transfers occurring within 24 h into a single admission. The total length of stay was calculated by summing the days spent in hospital during each episode of care.

Primary care use during the pre-RCT and RCT phases was captured using OHIP physician billing records, including outpatient visits with providers whose primary specialty was family medicine or general practice, as recorded within the ICES Physician Database. Multiple billings by the same physician for the same patient on the same date were counted as one visit. Residents were considered to have received homecare services if they had at least one recorded visit from a home and community support provider in the Ontario Homecare Database (15).

### 2.6 Outcome measures

The main health service utilization outcomes in this study were ED visits (overall and via ambulance), non-elective (urgent/emergent) hospital admissions, length of stay during those hospital admissions, and primary care visits. These building-level outcomes were evaluated using ICES administrative datasets for the 1 month preceding and 12-months during and following RCT. Further details regarding the data sources are provided above.

### 2.7 Statistical analysis

Frequencies and percentages were calculated for demographic variables. For the study outcomes, descriptive analyses were performed for the 1-month pre-RCT period, 12-month RCT period, and the 12-month post-RCT period for the CP@clinic and usual care groups. The means and standard deviations were standardized for a building of 100 residents.

For each health service utilization outcome measure, the CP@clinic and usual care groups were compared at the building-level using Generalized Estimating Equations (GEE) adjusted for building pairing and the building's baseline value for the same outcome measure during the pre-RCT month. All GEEs used a first-order autoregressive correlation structure.

As described above, during the RCT, it was discovered that two buildings did not meet the eligibility criteria. Therefore, *post hoc* sensitivity analyses were conducted using the same methods but excluding these two pairs of buildings (see Figure 1). All regression results are reported as adjusted incidence rate ratios (aIRR) with corresponding 95% confidence intervals (CI). Exact *p*-values have been reported and no corrections have been made (e.g., Bonferroni). All analyses were performed using SAS software, version 9.4 (SAS Institute Inc).

## 3 Results

### 3.1 Characteristics of residents

The study population included a total of 3,751 individuals, with 1,879 residents in the intervention/CP@clinic arm and 1,872 individuals in the control/usual care arm (Table 1). The pooled mean age across CP@clinic buildings, weighted by number of building residents, was 72.89 (SD = 8.74) and across usual care buildings was 72.26 (SD = 8.35). The majority of study residents were aged 65 years or older (77.74%), while 11.30% were aged 80 years or older, and 64.68% (*n* = 2,426) were female. According to the Johns Hopkins ACG Frailty Indicator, 594 (15.84%) were considered to be frail; 290 (14.37%) in the CP@clinic group, and 304 (16.24%) in the usual care group. See Supplementary Table S1 for the demographics of the sites included in the sensitivity analysis.

**Table 1:** Demographics of the complete study sample.

Measure	Total (*n* = 3,751)	CP@clinic (*n* = 1,879)	Usual Care (*n* = 1,872)
Age [years], *n* (%) <65 65–79 ≥80	835 (22.26%) 2,492 (66.44%) 424 (11.30%)	389 (20.70%) 1,262 (67.16%) 228 (12.13%)	446 (23.82%) 1,230 (65.71%) 196 (10.47%)
Female, *n* (%)	2,426 (64.68%)	1,275 (67.86%)	1,151 (61.49%)
ACG Frailty, *n* (%)	594 (15.84%)	290 (15.43%)	304 (16.24%)

### 3.2 Descriptive analyses

For building residents in the CP@clinic arm of the trial, the mean number of ED visits per month decreased from 9.49 per 100 residents (SD = 4.53) in the pre-RCT period to 9.25 per 100 residents (SD = 6.56) in the post-RCT period. In contrast, for the usual care arm, the monthly mean number of ED visits per 100 building residents increased from 7.49 (SD = 2.33) to 8.74 (SD = 4.94). This same pattern was observed for all other outcomes (Table 2). For descriptive analyses of the sites included in the sensitivity analysis, please see Supplementary Table S2.

**Table 2:** Description of building-level health service utilization per 100 residents in CP@clinic and usual care sites (1 building = unit of analysis, weighted equally).

Health Service Utilization Outcome	Period	Monthly Mean	
		CP@clinic Mean per 100 building residents (SD)	Usual Care Mean per 100 residents (SD)
ED visits	Pre-RCT period	9.49 (4.53)	7.49 (2.33)
	RCT period	9.37 (4.55)	8.40 (3.77)
	Post-RCT period	9.25 (6.56)	8.74 (4.94)
ED visits by Ambulance	Pre-RCT period	4.79 (2.31)	3.96 (2.51)
	RCT period	4.13 (2.78)	4.21 (2.69)
	Post-RCT period	4.10 (4.62)	4.39 (3.22)
Non-elective Hospital Admissions	Pre-RCT period	3.62 (3.40)	1.63 (1.40)
	RCT period	2.01 (1.63)	2.33 (1.78)
	Post-RCT period	1.82 (1.44)	2.25 (1.84)
Number of Days Admitted	Pre-RCT period	33.66 (42.35)	10.21 (9.84)
	RCT period	21.29 (27.47)	23.18 (30.36)
	Post-RCT period	19.22 (23.15)	19.59 (29.51)
Primary Care Visits	Pre-RCT period	64.72 (21.32)	54.41 (18.04)
	RCT period	56.49 (18.04)	54.11 (17.89)
	Post-RCT period	57.75 (18.77)	54.73 (18.77)

### 3.3 GEE modelling

After adjusting for baseline outcome event rates and building clustering, CP@clinic buildings had significantly lower hospital admission rates during the 12-month RCT period (aIRR = 0.75; 95% CI: 0.61–0.91) and in the 12 months following the RCT (aIRR = 0.82; 95% CI: 0.70–0.96) compared to usual care buildings. CP@clinic buildings had lower primary care visits during the RCT period (aIRR = 0.93; CI: 0.88-0.99), and higher visits in the post-RCT period (aIRR = 1.05; CI: 1.00-1.10). There was no significant difference in the rates of ED visits, ED visits by ambulance, and the number of days admitted to hospital between CP@clinic and usual care buildings during the 12-month RCT period or in the 12 months post-RCT (Table 3).

**Table 3:** Difference in rate of monthly health service utilization within 12-months during RCT and following post-RCT, between buildings receiving CP@clinic and usual care, adjusting for baseline and building clustering.

Health Service Utilization Outcome	Period	Adjusted^a^ Incidence rate ratio (aIRR)	*P*-value
ED visits	RCT period	1.00 (0.92, 1.09)	0.998
	Post-RCT period	1.07 (0.90, 1.28)	0.438
ED visits by Ambulance	RCT period	0.89 (0.76, 1.04)	0.134
	Post-RCT period	0.89 (0.69, 1.16)	0.404
Non-elective Hospital Admissions	RCT period	0.75 (0.61, 0.91)	0.005*
	Post-RCT period	0.82 (0.70, 0.96)	0.017*
Number of Days Admitted	RCT period	0.94 (0.71, 1.24)	0.656
	Post-RCT period	1.03 (0.86, 1.24)	0.760
Primary Care Visits	RCT period	0.93 (0.88, 0.99)	0.019*
	Post-RCT period	1.05 (1.00, 1.10)	0.031*

a Adjusted for building pairing and the building's baseline value for the same outcome.

*Significant at *P* < 0.05.

### 3.4 Sensitivity analysis

In the sensitivity analysis after removing the two building pairs, after adjusting for baseline outcome event rates and building clustering, ED visits by ambulance were significantly lower in CP@clinic buildings during the 12-month RCT period (aIRR = 0.81; 95% CI: 0.70–0.94), with no significant difference observed in the 12 months post-RCT (aIRR = 0.88; 95% CI: 0.65–1.18), compared to usual care (Table 4). Non-elective hospital admission rates also were significantly lower in the CP@clinic group during the RCT period (aIRR = 0.66; CI: 0.55-0.81) and in the post-RCT period (aIRR = 0.78; CI: 0.63-0.96), compared to usual care. Primary care visits were significantly lower in the CP@clinic buildings compared to usual care during the RCT period (aIRR = 0.92; CI: 0.85-0.99) but were significantly higher in the post-RCT period (aIRR = 1.05; CI: 1.00-1.10). There were no statistically significant differences observed in the number of ED visits and number of days admitted to the hospital between the CP@clinic and usual care buildings during the RCT and post-RCT periods.

**Table 4:** Sensitivity analysis of difference in rate of monthly health service utilization rates within 12-months during RCT and following 12 months post-RCT, between units receiving CP@clinic and usual care, adjusting for baseline and building clustering.

Health Service Utilization Outcome	Period	Adjusted^a^ Incidence rate ratio	*P*-value
ED visits	RCT period	0.97 (0.89, 1.05)	0.424
	Post-RCT	1.08 (0.89, 1.31)	0.430
ED visits by Ambulance	RCT period	0.81 (0.70, 0.94)	0.005*
	Post-RCT	0.88 (0.65, 1.18)	0.386
Non-elective Hospital Admissions	RCT period	0.66 (0.55, 0.81)	<0.001*
	Post-RCT	0.78 (0.63, 0.96)	0.018*
Number of Days Admitted	RCT period	0.79 (0.62, 1.02)	0.073
	Post-RCT	1.02 (0.83, 1.27)	0.835
Primary Care Visits	RCT period	0.92 (0.85, 0.99)	0.020*
	Post-RCT	1.05 (1.00, 1.10)	0.037*

a Adjusted for building pairing and the building's baseline value for the same outcome.

*Significant at *P* < 0.05.

## 4 Discussion

This study demonstrated health system-related relevant and meaningful reductions in non-elective hospital admissions during the RCT period that were sustained after the RCT period, along with an increase in primary care visits in the post-RCT phase. These findings are important as they suggest that the CP@clinic program may facilitate a shift in care from acute settings to more appropriate, community-based care. This result supports the value of the evidence-based community paramedicine program, CP@clinic, in improving access to preventive care and reducing the burden on acute care services, particularly in vulnerable populations (6, 7).

The observed reduction in non-elective hospital admissions, including a sustained effect post-RCT, highlights the potential of the CP@clinic program to produce lasting changes in health system use. In a study by Mbata et al., it was found that fewer hospitalizations could indicate better chronic disease management and earlier intervention in the community (16). This is in keeping with our findings from the primary RCT study which demonstrates that CP@clinic facilitates anti-hypertensive medication initiation (7). Similar effects have been seen in other preventive care models, such as in a study by Musich et al. utilizing physicians, which have also reported reductions in acute care use over time and demonstrated a sustained impact beyond one year (17). The potential continued impact of any preventative program, such as CP@clinic, has important implications for health systems as they will also aim to improve outcomes while reducing future health care costs (4).

The post-RCT increase in primary care visits observed in the current study is also a promising outcome as previous studies have found that shifts toward primary care reflect greater engagement with preventative services, medication management, and chronic disease monitoring (18). Literature supports this pattern, where greater use of primary care is associated with improved health outcomes, particularly for ambulatory care sensitive conditions, and reduced reliance on emergency services (18, 19). As individuals receive more timely care from family physicians, there is an opportunity to detect issues earlier, preventing escalation to requiring emergency care or hospitalization. Further research is needed to identify the ambulatory care sensitive condition-specific strategies that effectively reduce hospitalizations, particularly for high-risk conditions such as chronic obstructive pulmonary disease (19, 20). This reorientation toward primary care (21) is a critical marker of improved access, continuity, and appropriateness of care (22, 23), especially in underserved, vulnerable populations, such as those living in social housing.

While ED visits by ambulance were not significantly reduced in this study, even after sensitivity analyses, this finding must be interpreted in context. Our previous research has shown a reduction in ambulance call volume following CP@clinic implementation (6). It is possible that individuals who called 9-1-1 were not always transported if their condition was not severe, particularly if better community management and preventive care had already been provided. In a Canadian population-based study, 19% of ambulance calls did not result in transport to hospital (24). According to the Ministry of Health and Long-Term Care 2013 Annual Report of the Office of the Auditor General of Ontario, over 25% (or about 350,000) of ambulances dispatched did not transport a patient (25). Furthermore, among those who were transported, the acuity may have been lower since there were fewer hospital admissions, reflecting potentially improved overall health and better self-management of chronic conditions. Evidence shows that 63.7% of patients brought to Ontario emergency rooms by ambulance in 2019 were discharged without being admitted to the hospital (25). These points highlight the importance of distinguishing between ambulance arrivals, the clinical context, and the need for transport.

### 4.1 Strengths and limitations

This study offers several important strengths. One strength is its combination of a pragmatic cluster randomized trial and outcome evaluation leveraging unobtrusive, routinely collected administrative data, which eliminates the need for primary data collection. Implementing such a methodology is less popular in assessments of community interventions due to the inherent challenges involved in using cluster randomization and administrative data in real-world contexts (26). Furthermore, the study setting and outcomes based upon administrative health care data are highly relevant and broadly generalizable to other jurisdictions with publicly funded health care and social programs, such as social housing settings such as the UK, the US, and Australia. One potential limitation is potential for misclassification of social housing residents due to out-of-date addresses in the provincial health insurance registry caused by delays in reporting and processing updates. However, given this was a trial and control buildings were matched in time and by geographic location, we do not believe this is likely to introduce bias into our evaluation. The data we analysed in this study are now somewhat older, however we feel that the results are still very relevant as there has been little Canadian research using RCT methods to evaluate community paramedicine interventions. Despite COVID-19 and other health system changes, these results are still informative for future research and clinical practice and can be a comparison. Finally, during the post-RCT period, the implementation was pragmatic and determined by the paramedic services, not the research team. While there were varying frequencies of CP@clinic sessions in the buildings, the RCT study was not designed or powered to compare these differences. Future research is needed to specifically examine the varying frequency of CP@clinic sessions and how this impacts health care utilization outcomes to determine opportunities for targeted approaches and the reduction of session frequency over time.

## 5 Conclusions

In conclusion, the CP@clinic program was associated with a sustained reduction in hospital admissions and an increase in primary care visits beyond the RCT time period, suggesting a shift from reactive to preventive care among social housing residents. Although this analysis did not show a significant reduction in ED visits by ambulance, our previous research indicates a broader decline in emergency service use at the level of 9-1-1 calls for both transports and non-transports (6). The lasting impact beyond the RCT period suggests that community paramedicine programs may improve and have lasting effects on health system efficiency and primary care access, and reductions in health inequities in vulnerable populations.

## Author's note

This document used data adapted from the Statistics Canada Postal Code^OM^ Conversion File, which is based on data licensed from Canada Post Corporation, and/or data adapted from the Ontario Ministry of Health Postal Code Conversion File, which contains data copied under license from ^©^Canada Post Corporation and Statistics Canada. Parts of this material are based on data and/or information compiled and provided by the Canadian Institute for Health Information and the Ontario Ministry of Health.

## Data Availability

The datasets presented in this article are not readily available because the data that support the findings of this this study are held securely in coded form at ICES. Although legal data sharing agreements between ICES and data providers (e.g., health care organizations and governments) prohibit ICES from making the dataset publicly available, access may be granted to those who meet prespecified criteria for confidential access, available at https://www.ices.on.ca/DAS (das@ices.on.ca). The full dataset creation plan and underlying analytical code are available from the authors upon request, understanding that the computer programs may rely upon coding templates or macros that are unique to ICES and are therefore either inaccessible or may require modification. Requests to access the datasets should be directed to the corresponding author.
