# Supplementary material for: Effect of the CP@clinic program (Community Paramedicine at Clinic program) on health care utilization from administrative data during and after the intervention: a cluster-randomized controlled trial
Source: Front Health Serv. 2026 Oct 15;6:1939050. doi: 10.3389/frhs.2026.1939050 (PMC13716780; doi:10.3389/frhs.2026.1939050)
Supplement: Supplementary file 1 [file Table1.docx]

**Table S1: Demographics for residents of the sensitivity analysis buildings**

|  | **Total (n = 3603)** | **CP@clinic (n=1779)** | **Usual Care (n=1824)** |
| --- | --- | --- | --- |
| **Age [years], n (%)**  **<65**  **65 - 79  ≥80** | 738 (20.48%)  2448 (67.94%)  417 (11.57%) | 322 (18.10%)  1233 (69.31%)  224 (12.59%) | 416 (22.81%)  1215 (66.61%)  193 (10.58%) |
| **Female, n (%)** | 2343 (65.03%) | 1217 (68.41%) | 1126 (61.73%) |
| **ACG Frailty, n (%)** | 579 (16.07%) | 280 (15.74%) | 299 (16.39%) |
