# Supplementary material for: Effect of the CP@clinic program (Community Paramedicine at Clinic program) on health care utilization from administrative data during and after the intervention: a cluster-randomized controlled trial
Source: Front Health Serv. 2026 Oct 15;6:1939050. doi: 10.3389/frhs.2026.1939050 (PMC13716780; doi:10.3389/frhs.2026.1939050)
Supplement: Supplementary file 2 [file Table2.docx]

**Table S2: Health service utilization per 100 units in CP@clinic and usual care sites;**

**(1 building = unit of analysis, weighted equally)**

| **Health Service Utilization Outcome** | **Period** | **CP@clinic**  **Mean (SD)** | **Usual Care**  **Mean (SD)** |
| --- | --- | --- | --- |
| ED visits | Pre-RCT period | 9.40 (4.60) | 7.40 (2.50) |
|  | RCT period | 9.19 (4.75) | 8.41 (3.84) |
|  | Post-RCT period | 9.03 (6.92) | 8.55 (4.97) |
| ED visits by Ambulance | Pre-RCT period | 4.96 (2.34) | 4.07 (2.67) |
|  | RCT period | 3.91 (2.80) | 4.27 (2.73) |
|  | Post-RCT period | 3.84 (4.83) | 4.29 (3.28) |
| Non-elective Hospital Admissions | Pre-RCT period | 3.67 (3.67) | 1.58 (1.50) |
|  | RCT period | 1.90 (1.68) | 2.44 (1.82) |
|  | Post-RCT period | 1.68 (1.44) | 2.17 (1.78) |
| Number of Days Admitted | Pre-RCT period | 34.84 (45.59) | 10.20 (10.59) |
|  | RCT period | 19.70 (27.59) | 24.47 (31.44) |
|  | Post-RCT period | 17.59 (22.63) | 18.81 (29.23) |
| Primary Care Visits | Pre-RCT period | 68.64 (20.13) | 54.68 (19.21) |
|  | RCT period | 58.36 (18.49) | 54.29 (18.62) |
|  | Post-RCT period | 58.84 (19.79) | 55.16 (19.78) |
